# The Role and Mechanism of Essential Selenoproteins for Homeostasis

**DOI:** 10.3390/antiox11050973

**Published:** 2022-05-15

**Authors:** Ruihua Ye, Jiaqiang Huang, Zixu Wang, Yaoxing Chen, Yulan Dong

**Affiliations:** Key Laboratory of Precision Nutrition and Food Quality, Ministry of Education, College of Veterinary Medicine, China Agricultural University, Beijing 100193, China; yerh0522@cau.edu.cn (R.Y.); jqhuang@cau.edu.cn (J.H.); zxwang@cau.edu.cn (Z.W.); yxchen@cau.edu.cn (Y.C.)

**Keywords:** selenoproteins, oxidation resistance, Se-enriched food, homeostasis

## Abstract

Selenium (Se) is one of the essential trace elements that plays a biological role in the body, mainly in the form of selenoproteins. Selenoproteins can be involved in the regulation of oxidative stress, endoplasmic reticulum (ER) stress, antioxidant defense, immune and inflammatory responses and other biological processes, including antioxidant, anti-inflammation, anti-apoptosis, the regulation of immune response and other functions. Over-loading or lack of Se causes certain damage to the body. Se deficiency can reduce the expression and activity of selenoproteins, disrupt the normal physiological function of cells and affect the body in antioxidant, immunity, toxin antagonism, signaling pathways and other aspects, thus causing different degrees of damage to the body. Se intake is mainly in the form of dietary supplements. Due to the important role of Se, people pay increasingly more attention to Se-enriched foods, which also lays a foundation for better research on the mechanism of selenoproteins in the future. In this paper, the synthesis and mechanism of selenoproteins, as well as the role and mechanism of selenoproteins in the regulation of diseases, are reviewed. Meanwhile, the future development of Se-enriched products is prospected, which is of great significance to further understand the role of Se.

## 1. Introduction

**Figure 1:**
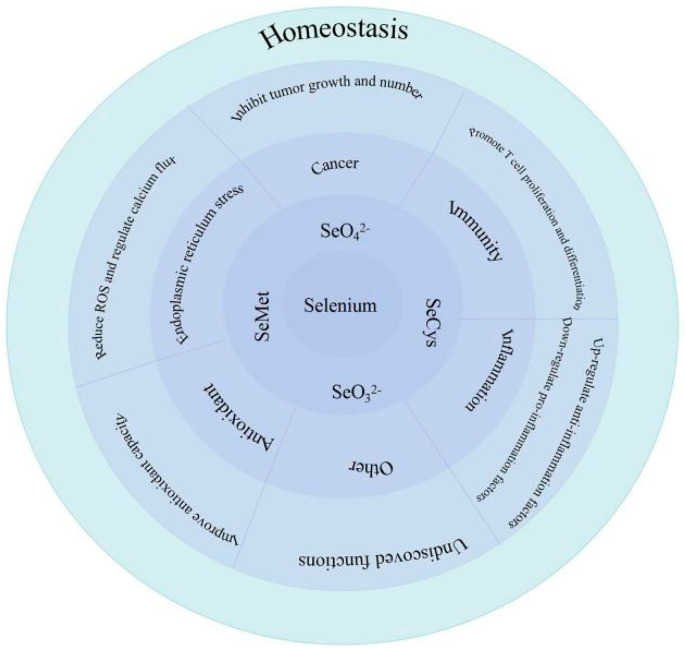
Biological role of Se.

Selenium (Se), an essential trace element, was first discovered in 1817 and its physiological functions were gradually excavated [1]. Se level is necessary for maintaining homeostasis, including muscle function, male reproductive biology, cardiovascular function, endocrine, the nervous system and especially the immune system [1,2]. The main role of Se can be observed in Figure 1. Se is widely distributed in soil, water, plants, fruits, vegetables, meat, eggs and milk. Dietary Se can be obtained from foods, such as grains, nuts, vegetables, fish, meat, dairy and poultry products [3,4]. Although Se has a good biological activity, amounts that are too low or too high in the body cause adverse effects [2]. Studies have shown that Se deficiency can cause Keshan disease (KD) and Kashin–Beck disease (KBD) [5]. KD is characterized by acute heart failure, congestive heart failure, and arrhythmia. It can also be divided into acute, subacute, chronic and latent KD [6]. KBD can lead to permanent and disabling deformities, including muscular atrophy, hyperplasia, dyskinesia, waddling gait, canard walking, joint linkage, short fingers, short limbs and short stature in some adults. Se supplementation can prevent KD and KBD [7]. However, high Se level can be toxic, engendering symptoms, such as hair loss; brittle, thickened and layered nails; garlicky breath and nervous system abnormalities [1,2].

Se, as a trace element, usually enters the body in the form of organic Se and inorganic Se. Inorganic Se is generally selenate, selenite. Organic Se is selenomethionine (Se-Met) and selenocysteine (Sec) [8,9]. Sec and Se-Met play biological roles in the body. Sec is a translation behavior mediated by codon UGA, and Se-Met binds to protein through methionine. Generally, the proteins formed by Se in the form of Sec are called selenoproteins, while the other forms of protein combined with Se are called se-containing proteins [10]. Selenoproteins can be involved in oxidative stress, endoplasmic reticulum stress and the regulation of immune and hormone levels to affect homeostasis [11]. The expression of selenoproteins is also closely related to the occurrence and development of some diseases [2,10].

Considering the outstanding biological activity of Se, several studies have revealed that Se is involved in regulating the occurrence of many diseases [2]. In addition, there are regional differences in the distribution of Se, so it is particularly important to improve Se level in the body through exogenous Se supplementation in Se-deficient areas [1,3]. Therefore, in addition to studying the specific mechanism of Se and selenoproteins regulating diseases, researchers are gradually focusing on the development of Se-enriched food [12]. Dietary Se supplements are an important method for Se supplementation; researchers have developed many Se-enriched foods, such as Se-enriched rice, Se-enriched apples and Se-enriched eggs with reasonable Se concentrations [12,13,14]. To a certain extent, it is also of great significance for the future research on the rational application of Se and selenoproteins metabolism and physiological functions to improve human and animal health.

The Se element is crucial to organisms. The common forms of Se are selenate, selenite, Sec and Se-Met. Se can resist external stimuli through a variety of signaling pathways to maintain hemostasis. Se can resist external stimuli through a variety of signaling pathways and maintain health, which are mainly reflected in promoting T-cell proliferation and differentiation to play a role in regulating immunity, inhibit the growth and number of tumors for an anticancer effect, regulate the level of inflammatory factors to attain an anti-inflammatory function, reduce the production of ROS and regulate calcium flux to resist ER stress and improve antioxidant capacity to play an antioxidant role. However, due to the limited research on Se, there are still some unrevealed effects of Se, and the potential effects will be discovered in future studies.

## 2. Synthesis and Metabolism of Selenoproteins

### 2.1. Diet Sources and Metabolism of Se in the Body

**Figure 2:**
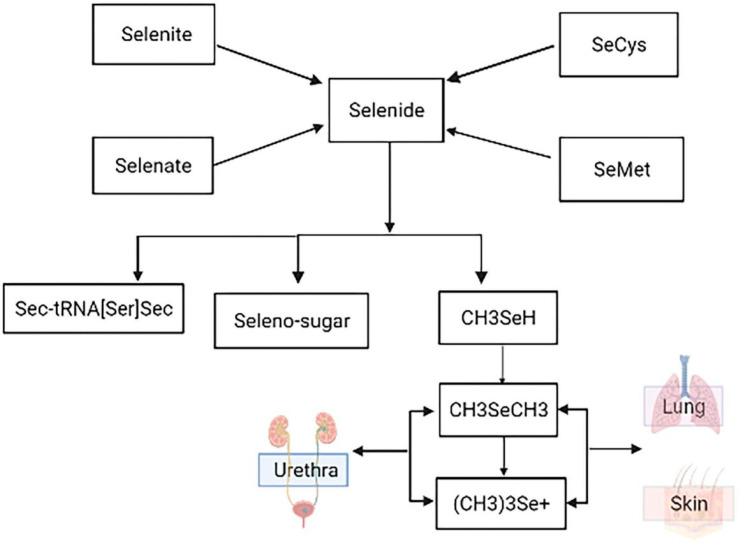
Metabolism of Se element.

Se, as an essential trace element, plays an important role in the body. The main sources of Se are bread, cereals, eggs, meat, fish, dairy products, fruits and vegetables [1,7,9]. Studies show that the content of Se in food is low and the form of Se is more complex. The qualitative and quantitative analyses of the form of Se in food mainly focus on selenate, selenite, Se-Met, Sec, selenomethylselenocysteine, but the forms of Se in food are far more than these [2,7]. Se consumed by humans through diet is mainly obtained from plants, which absorb various forms of Se from the soil through the roots, and then transform them into organic forms through metabolism, such as Se-Met and Sec amino acids and selenoproteins [4,8,9]. The main absorption site of Se is the small intestine. Se can enter the body through the respiratory tract and skin, and its main absorption site is the small intestine [10]. Se is absorbed by red blood cells soon after intestinal absorption. Through a series of reduction reactions involving glutathione and glutathione reductase, Se is reduced to hydrogen selenide, which becomes the active Se source in selenoprotein synthesis [8]. There are also some differences in the absorption modes of different Se sources. Selenite is absorbed by simple diffusion; organic Se follows the amino acid absorption mechanism [10,15,16]. Se is reduced to hydrogen selenide through a series of reduction reactions involving glutathione and glutathione reductase. Hydrogen selenide is the active Se source in selenoprotein synthesis, and then selenoprotein synthesis is transported to different tissues and organs by the blood for biological activity [16,17,18]. When Se in the body is insufficient, the use of accumulated selenoproteins is preferred. At the same time, under the action of continuous methylation, hydrogen selenide can generate dimethyl and trimethyl Se ions [19]. When the body absorbs excessive Se, it can be metabolized into volatile dimethyl Se and trimethyl Se compounds, which can be excreted through the lung, resulting in garlic breath. Se is excreted from the body mainly through the urine and kidneys, but also in small amounts through feces, sweat and hair [1,8,19]. The detailed metabolic process can be observed in Figure 2.

Se sources (inorganic Se: sodium selenite, selenate; organic Se: Sec, Se-Met) are first converted into selenides for utilization in organisms. Subsequently, selenides are then converted into other forms to be absorbed and used by the body. It is mainly absorbed by the body in the form of selenoproteins, and part of it can be converted into Se-sugar. The unabsorbed selenide forms methyl selenide through methylation, and dimethyl and trimethyl compounds are generated with continuous methylation, which are discharged from the body through respiration, perspiration, urine and feces.

### 2.2. Se-Synthesis of Selenoproteins

**Figure 3:**
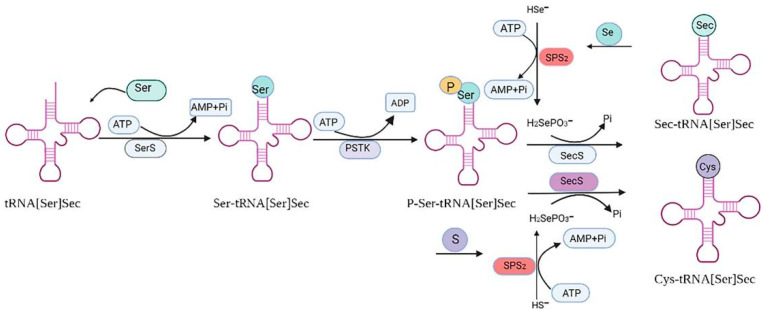
Synthesis of selenoproteins.

Se can enter the body through various forms of compounds, but is mainly absorbed and utilized in the form of selenoproteins [3]. To date, 25 selenoproteins have been isolated and tested in humans, and 25 selenoproteins have been identified, including 5 glutathione peroxidases (GPXs), 3 thioredoxin reductases (TrxRs), 3 iodothyronine deiodinases (DIOs), selenium phosphorylate synthetase (SPS), selenoprotein H (SELENOH), selenoprotein O (SELENOO), selenoprotein P (SELENOP), selenoprotein T (SELENOT), selenoprotein W (SELENOW), selenoprotein N (SELENON), selenoprotein M (SELENOM), selenoprotein S (SELENOS), selenoprotein I (SELENOI), selenoprotein K (SELENOK), selenoprotein 15 (15kDa), selenoprotein R (SELENOR) and selenoprotein V (SELENOV) [3,17,20]. Se is mainly present in organisms in the form of Sec and Se-Met, which act by binding with proteins. Sec is the 21st naturally occurring amino acid discovered in living organisms [20,21]. It is encoded by the stop codon UGA, and under specific translation conditions [16]. Sec can be transferred to the synthetic selenoprotein polypeptide chain by selenocysteine-specific-transport RNA (tRNAsec). First, the RNA of transported selenocysteine (tRNA[Ser]Sec) was combined with serine and acylated to form Ser-tRNA[Ser]Sec under the action of Serine-tRNA synthase (SERS). Ser-tRNA[Ser]Sec was then phosphorylated by kinase (PSTK) to form pSer-tRNA[Ser]Sec. Finally, Sec synthase (SPS2) catalyzed the formation of Ser-tRNA[Ser]Sec from pSer-tRNA[Ser]Sec and the Se donor H_2_SePO^3−^ [15,18,21]. Se-Met can be utilized in two ways in the body. Firstly, Se-Met is converted into selenocysteine through the sulfur-conversion pathway to participate in selenoprotein synthesis. Secondly, Se-Met is degraded to methyl selenol by cysteine-γ lyase in vivo, which is converted to dimethyl selenide/trimethyl selenide by methylation with S-adenosine methionine, and then converted to selenide by demethylation to participate in selenoprotein synthesis [5,15,18]. The process of selenoprotein synthesis is detailed in Figure 3.

First, the RNA-transporting selenocysteine (tRNA[Ser]Sec) was acylated with seric acid under the action of Ser-tRNA synthase (SERS) to form Ser-tRNA[Ser]Sec. Ser-tRNA[Ser]Sec was then phosphorylated by kinase (PSTK) to form pSertRNA[Ser]Sec. Finally, Sec synthase (SPS2) promoted pSertRNA[Ser]Sec to form SectRNA[Ser]Sec with the Se donor H_2_SePO^3−^. When Se was replaced with sulfur, Sec synthase (SPS2) promoted pSertRNA[Ser]Sec and H_2_SePO^3−^ to produce Cys-tRNA[Ser]Sec.

## 3. Selenoproteins Regulate Related Diseases

### 3.1. Selenoproteins and Cardiovascular Diseases

Se deficiency was first linked to KD, an endemic disease characterized by cardiomyopathy and heart failure [19]. Se deficiency is associated with a variety of cardiovascular diseases, including cardiomyopathy, such as KD, heart failure and myocardial infarction, cardiomyopathy, atherosclerosis and coronary heart disease [22]. Several selenoproteins have been shown to be associated with cardiovascular disease. GPx1 is a stress-responsive selenoprotein, meaning it is tightly regulated by Se levels. Se deficiency may help to down-regulate GPx1 expression, reducing the heart’s ability to respond to oxidative stress and leading to poor cell survival [23]. The primary function of DIO is to regulate thyroid hormone levels, which attenuates cardiac remodeling after myocardial infarction. Reduced plasma levels of the thyroid hormone are associated with heart failure and increased DIO3 expression [24,25]. TrxR mainly regulates intracellular redox reactions and participates in DNA synthesis, immune response and apoptosis in mammals. TrxR, which is subdivided into TrxR1, TrxR2 and TrxR3, plays a role in reducing oxidative stress to hypertrophy caused by stress overload and improving left ventricular remodeling [26,27]. T. A. et al. showed that SELENOT prevented the free-radical damage of cell death during ischemia/reperfusion. SELENOT-derived peptides protect the heart from ischemia-reperfusion injury by inhibiting apoptosis and oxidative stress [28]. ER stress is an important factor in regulating cardiac apoptosis/survival decisions during various stress processes. SELENOK is also associated with SELENOS regulating ER stress induced by misfolded proteins. In addition, SELENOM, SELENON and Sep15 are present in ER and may regulate cardiac response to ER stress [29,30,31].

### 3.2. Selenoproteins and Liver Disease

Non-alcoholic fatty liver disease (NAFLD) is often complex and often associated with serious complications, such as obesity and/or insulin resistance, and has become the most common chronic liver disease in the world. High-plasma Se and SELENOP levels are associated with insulin resistance and NAFLD, the SELENOP level was positively correlated with insulin resistance and NAFLD, but for serum Se, the conclusions were different [32,33]. An. et al. showed that adding 1.0 mg/kg of Se can reduce the liver injury induced by high fat [34]. The mRNA level of SELENOS in the liver of pigs induced by high fat can be significantly increased, and the expression of SELENOS is negatively correlated with the trend of apoptosis rate and symptoms of non-alcoholic steatohepatitis. This also suggests that SELENOS may be essential in the protection of liver damage induced by high fat [34,35]. The liver, as the central organ of Se regulation, regulates the Se level of the whole body by forming SELENOP and then transports it to other organs [33]. Studies have shown that dietary Se deficiency can reduce liver selenase activity and lead to oxidative stress, and activate oxidative stress-related signals through [36,37]. Se deficiency induces redox imbalance by regulating selenoproteins at mRNA and protein levels, thereby blocking the GSH system while enhancing GSH synthesis and catabolism [37]. Se and selenoproteins play an immunomodulatory role in hepatocellular carcinoma (HCC) by regulating oxidative stress, inflammation, immune response, cell proliferation and growth, angiogenesis, signaling pathways and apoptosis [36,38]. Sang et al. showed that Se concentration was usually low in the tissues of HCC patients, and Se concentration in the tissues of HCC patients could be higher by exogenous Se supplementation than before, thus reducing the number and size of tumors [38].

### 3.3. Selenoproteins and Brain Diseases

Most expression of selenoproteins are proven in the brain, especially in cortex and hippocampus neuron dysfunction [39,40]. Studies have found that selenoproteins can lead to impaired cognitive function and neural systemic disease [41,42,43]; existing research has shown that selenoproteins in brain diseases, such as Alzheimer’s disease (AD), Parkinson’s disease (PD) and Huntington’s disease (HD), play an important role in epilepsy [44,45,46]. Selenoproteins are important for normal brain function, and a decline in their function can lead to impaired cognitive function and neurological diseases [40,43]. Se deficiency has been associated with cognitive decline, and selenoproteins may help prevent the neurodegeneration of AD [45]. PD is associated with the impaired function of glutathione peroxidase selenase [46]. In HD, Se prevents lipid peroxidation by increasing specific glutathione peroxidase enzymes [47]. Se deficiency can increase the risk of seizures, and Se supplements may help alleviate them. SELENOP plays a crucial role in Se homeostasis in the brain, and GPx1 and GPx4 are the main forms of GPx in the brain. GPx4 functions at different sites in neurons, including cytosol, mitochondria and the nucleus [41,43,47]. GPx4 destroys phospholipid hydroperoxides and, together with vitamin E, inhibits lipid peroxidation in various cell membranes and lipoproteins. GPx4 has a potential role in AD, epilepsy and HD [45,46,47]. TrxR related to brain function is mainly TrxR1 and TrxR2. They reduce hydrogen peroxide and oxidative stress, and regulate redox sensitive transcription factors that control cellular transcription mechanisms. The protein family may have a protective role in AD and epilepsy [39,41,43,48].

### 3.4. Selenoproteins and Intestinal Diseases

Gastrointestinal diseases have become one of the most important diseases threatening global health, and there is no complete cure at present, so the research on intestinal tracts is particularly urgent [49]. A large number of recent studies have linked Se levels to the incidence and severity of intestinal diseases, such as inflammatory bowel disease (IBD) and colorectal cancer (CRC) [50]. Se alleviates intestinal inflammation through the action of selenoproteins, which play a protective role in intestinal infection by strengthening type-3 innate lymphocytes (ILC3) and helper T-cells 17 (Th17), which are required by the intestinal epithelial barrier [51,52]. The intestinal barrier is necessary to maintain intestinal function. There are many factors that can lead to intestinal barrier damage [53], among which inflammation can increase the production of ROS and damage the intestinal barrier, while dietary Se supplementation can reduce the level of ROS [54]. With the antioxidant function of selenoproteins that can play roles in intestinal inflammation, spontaneous colitis can be induced in GPx1 and GPx2 knockout mice [55]. In vitro and in vivo studies of Sep15 knockout colon cancer cells or mouse models using systemic knockout Sep15 observed a reversal of colon cancer phenotype and a reduction in the number of chemically induced tumors, respectively [56,57]. SELENOP, one of the most important selenoproteins, is significantly reduced in the serum of CD, and serum SELENOP concentration is negatively correlated with CRC risk [22]. C.S.W. et al. demonstrated that SELENOP of colonic epithelial origin is the source of antioxidant-mediated cancer protection associated with colitis, and that SELENOP down-regulation promotes oxidative stress in ulcerative colitis (UC) [58]. Intestinal epithelial conditional deletion increases tumor load and genomic instability in the CAC model, suggesting an important role of SELENOP in colon cancer development [51,58]. In addition, reduced Se levels promote T-assisted (Th1) cell differentiation in CD patients. Se supplementation can inhibit Th1 cell differentiation through SELENOW, and significantly remove cytoplasmic ROS, and relieve symptoms of patients with CD [59].

### 3.5. Selenoproteins and Cancer

Many studies have revealed that selenoproteins are closely related to the occurrence of tumor and the progression of cancer [3,20,22]. CRC is one of the most common cancer diseases and one of the most common causes of death. It is well known that both genetic and environmental factors contribute to the increased incidence and mortality from CRC [60,61]. As a kind of important selenoprotein, GPx can play a role in a variety of cancers [62,63]. The down-regulation of GPx1 can promote the development of breast, lung and head and neck cancers. The down-regulation of GPx1, GPx3 and SELENOP is associated with the tumogenesis of colon cancer [51,58,62,63]. As an important ER-resident protein, Sep15 can be highly expressed in a variety of organs, and it is down-regulated in liver, prostate, breast and lung cancers [64,65,66,67,68]. SELENOP, as the main selenoprotein in plasma, plays an important role in the development of colon cancer, and the down-regulation of SELENOP can promote the occurrence of colorectal cancer [58]. SELENOK negatively regulates human chorionic gonadotropin β subunit (β-HCG expression) and acts as a tumor suppressor in human choriocarcinoma cells, which may be a novel therapeutic target for human choriocarcinoma in vitro [69]. SELENOK can inhibit cell adhesion and the migration of human gastric cancer cells. SELENOK is critical in promoting calcium fluxes that induce melanoma progression [70,71]. The up-regulation of GPx2 and Sep15 was observed in bladder tumors and bladder cancer cells [59]. Due to the limited research on selenoproteins, the relationship between selenoproteins and cancer has not yet been revealed.

### 3.6. Selenoproteins and Reproduction

Se has a significant effect on the enzyme system of the male reproductive tract, and a lack of Se can affect testicular growth and delayed puberty [3]. Se plays an important role in sperm maturation in mammals, and Se deficiency can block sperm maturation [72]. Se level can affect semen quality and fertility; the process of sperm maturation affects male reproduction ability, so the Se level is necessary in male reproduction [73,74]. Se, as a component of selenoproteins and selenase, is involved in spermatogenesis by protecting sperm from ROS [73]. Selenoprotein knockout studies have shown that a lack of selenoproteins during spermatogenesis can lead to sperm abnormalities, which, in turn, affects semen quality and fertility [72]. Therefore, appropriate Se levels in the body are crucial for maintaining male reproductive function and avoiding infertility [71,72]. Se deficiency can reduce levels of selenoproteins involved in redox regulation, impair placental function and fetal development, and lead to miscarriage or complex preterm birth [73]. Thyroid hormone is essential for mammalian reproduction and growth [74]. DIO regulates thyroid hormone synthesis, and inadequate thyroid hormone synthesis has been associated with decreased fertility, disrupted estrus cycles, implantation dysfunction and uterine structural defects in humans and rodents, as well as other problems related to pregnancy health [73,75]. Selenoproteins are highly expressed in female reproductive tissues and play a role in follicular development and ovarian function [76].

**Figure 4:**
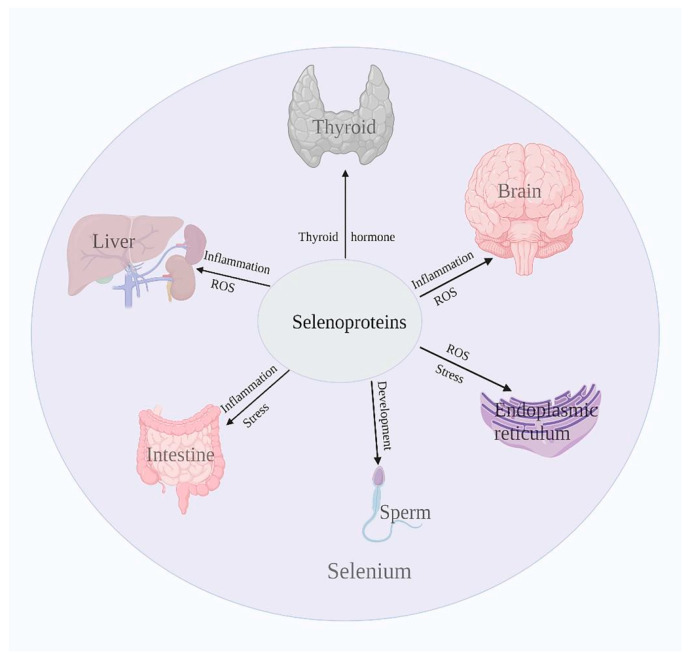
Selenoproteins regulate the health of many organs.

Selenoproteins can regulate homeostasis by regulating multiple tissues and organs in the body, and its participation in the regulation of major organs can be observed in Figure 4.

Selenoproteins are involved in the regulation of multiple tissues and organs to maintain homeostasis. DIO can regulate thyroid hormone levels, and decreased plasma levels of the thyroid hormone are associated with increased DIO3 expression. ER-resident proteins in selenoproteins can regulate ER oxidative stress and alleviate the damage caused by oxidative stress. Selenoproteins can also regulate intestinal health by regulating the production of reactive oxygen species and inflammatory response, and alleviate the development of diseases, such as in the nervous system. In addition, selenoproteins are also important in cancer and reproduction. Selenoproteins can affect the maturation process of sperm, which affects sperm motility and thus fertility. Moreover, many studies have shown that selenoproteins are closely involved in the development of cancer.

## 4. Mechanisms That Show That Selenoproteins Regulate Disease

Selenoproteins can be involved in regulating cellular oxidative stress, ER stress, antioxidant defense, immune response, inflammatory response and other biological processes in the body [1,3,22,74], including antioxidant, anti-inflammation, anti-apoptosis, and regulating immune response and other functions. Selenoproteins regulate body health in various and complex ways [5,18,20].

### 4.1. Selenoproteins Regulate Immune Responses

Immune responses are often closely related to inflammatory processes, and are also associated with ROS production and redox processes [20]. Se and selenoproteins regulate inflammatory and immune processes through redox function [77]. White blood cells, such as lymphocytes, macrophages and neutrophils, require ROS and pro-inflammatory molecules for activation, differentiation and phagocytosis [2]. The production of ROS increases the expression of inflammatory cytokines by increasing NF-κB activity. Because selenoproteins may affect these signaling pathways, they are also important in regulating immune responses and inflammation [78,79,80]. SELENOK is essential for calcium flux, T-cell proliferation and neutrophil migration in immune cells and has been shown to protect cells from ER stress-induced apoptosis [34]. Graves’ disease (GD) and Hashimoto’s thyroiditis (HT) are the most common autoimmune thyroid diseases (AITDs) [77]. Serum Se concentration in patients with autoimmune thyroid disease is reduced, and Se supplementation in patients with AITD can change inflammation and immune response to a certain extent. The main mechanisms involved include enhanced plasma GPX and TrxR activity and reduced toxicity of hydrogen peroxide and lipid hydroperoxides [78]. The researchers also found that MsrB1 controls immune responses by promoting the expression of anti-inflammatory cytokines in macrophages [80]. P.R. H. et al. found that SELENOK plays an important non-enzymatic role in regulating immunity as a cofactor of enzymes involved in key post-translational modifications of proteins. It also has a high catalytic efficiency and can play a biological role through antioxidant and protein repair [81]. The thymus and spleen are major reservoirs of T lymphocytes that regulate innate immune responses and provide protection against pathogens and tissue damage [82]. Feng et al. increased antioxidant capacity and selenoprotein expressions in offspring thymus and spleen through the maternal exogenous supplementation of organic Se sources, and reduced inflammation, autophagy and endoplasmic reticulum stress levels in offspring thymus and spleen [83].

### 4.2. Selenoproteins Reduce Inflammation

GPx1, as one of the important antioxidant enzymes in the body, can reduce the accumulation of pro-inflammatory factors and enhance the antioxidant capacity of the body, and its expression is affected by the Nrf2/ARE pathway [84]. ROS is produced under intracellular aerobic conditions and participates in cell proliferation, differentiation, apoptosis and other physiological activities. The main function of GPx is to remove ROS, including superoxide anions, hydrogen peroxide and hydroxyl radicals, and regulate the balance of intracellular redox [85]. GPx can catalyze glutathione in a variety of tissue cells, reduce peroxide to the corresponding alcohol, reduce oxidative stress and DNA oxidative damage, prevent free radicals from producing peroxidation and thus reduce the incidence of cell mutation [17,22]. Studies have shown that dietary Se supplementation can improve the activities of antioxidant enzymes, such as GPx and SOD, reduce the level of MDA and reduce DNA damage and cell apoptosis caused by oxidative stress [83,84]. GPx2 and GPx1 are important regulatory factors of epithelial cells and may affect inflammatory responses, and speculate that GPx1 and GPx2 have certain protective functions in colitis and inflammation-driven carcinogenesis [55,62,86]. Se and selenoproteins affect the immune response and epithelial barrier integrity after intestinal infection, mainly by regulating the mechanisms of ILC3 and Th17 cells to reduce inflammation and infection [51,52]. Se deficiency can target vascular tissue and mediate vascular injury through multiple pathways, such as necrosis, apoptosis and inflammation [85,87]. In vascular endothelial cells, increased selenoproteins activity may play a protective role by reducing abnormal cell adhesion induced by pro-inflammatory cytokines [30,31]. Additionally, the down-regulation of SELENOS under Se deficiency can effectively prevent the development of cardiovascular diseases, such as atherosclerosis and hypertension [87]. Selenoproteins protect the heart by damaging cholesterol that accumulates in blood vessel walls, increasing levels of coenzyme A in the heart muscle and increasing energy production [29].

### 4.3. Selenoproteins Inhibit ER Stress

ER is widely distributed in eukaryotic cells and plays an important role in protein processing, modification and steroid synthesis [87]. When there are too many unfolded or misfolded proteins in RE for a long period of time, calcium homeostasis imbalance can lead to an ER stress response. If it is not regulated, the ER activates the corresponding signaling pathway and induces apoptosis [88]. A variety of selenoproteins have an ER-response excitation adjustment function. Resident selenoproteins that regulate ER stress include 15 kDa selenoproteins, DIO2, SELENOS, SELENON, SELENOK, SELENOM and SELENOT [28,57,89,90]. ER-resident selenoproteins are involved in oxidative and ER stress, inflammation and/or intracellular Ca^2+^ homeostasis [91,92]. ER-resident selenoproteins are involved in homeostasis by regulating Ca^2+^ flux. SELENON appears to act as a redox cofactor for raniline receptors [89], while the redox enzyme Sep15 is associated with proteins involved in protein-folding quality control [56]. DIO2 is involved in thyroid hormone metabolism and also catalyzes redox reactions [8]. Future research will further reveal the mechanism of action of ER-resident proteins.

**Figure 5:**
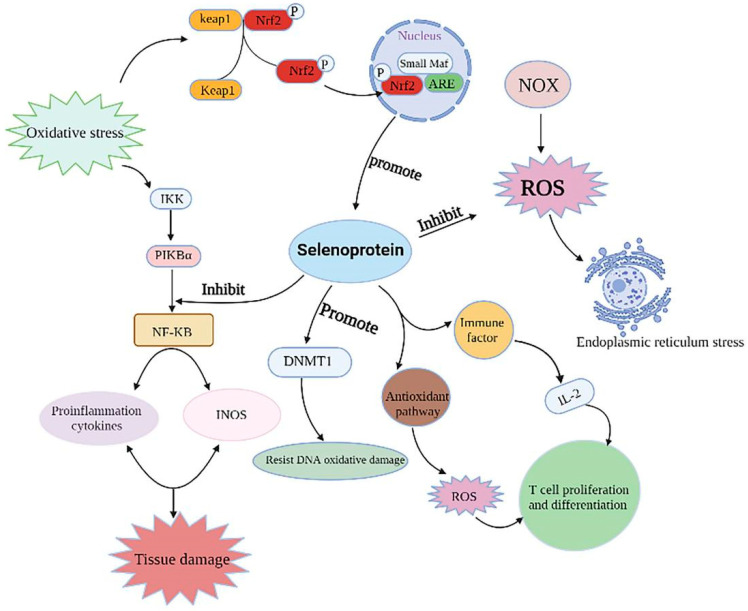
Selenoproteins regulate disease-related signaling pathways.

Selenoproteins play biological roles by regulating a variety of signaling pathways, and the detailed regulatory mechanisms can be observed in Figure 5.

GPx1, as one of the important selenoproteins, can reduce the accumulation of pro-inflammatory factors and enhance the antioxidant capacity of the body, and its expression is affected by the Nrf2/ARE pathway. When the body is subjected to oxidative stress, Nrf2 uncouples from the Keap1 protein, enters the nucleus and binds to ARE, activates the Nrf2/ARE pathway, enhances downstream GPx1 gene expression and alleviates oxidative stress. The expression of selenoprotein can reduce the expression of inflammatory factors, reduce the phosphorylation levels of IκK, IκBα and NF-κB P65, inhibit the production of pro-inflammatory factor NO and alleviate the pro-inflammatory response caused by oxidative stress. NADPH oxidase (NOX) can mediate excessive ROS production and induce ER oxidative stress, while selenoprotein can inhibit ROS production and relieve ER stress. In addition, selenoproteins can improve the antioxidant capacity of the body and cells by up-regulating the expression of DNA Methyltransferase 1 (DNMT1), block DNA oxidative damage and alleviate the toxic effect of cells. Selenoproteins maintain homeostasis through the regulation of various disease-related pathways.

## 5. Future Development Trend of Se and Selenoproteins

### 5.1. Se-Enriched Food Industry Develops Vigorously

Se is an essential trace element in the diet and is necessary for health and growth. It has been proved that the bioavailability of organic Se is higher than that of inorganic Se [1,8,10]. With the continuous improvement of living standards, people pay increasingly more attention to health. Considering the powerful antioxidant, anti-inflammatory and anti-cancer functions of Se, as well as the high biological activity of organic Se [6,20,22], the development and commercialization of organic Se reveals a new era of Se-enriched products. The existence of Se-deficiency areas makes the exogenous Se supplement particularly important; the intake of Se-enriched food (meat, milk and eggs) can be used to improve Se levels in the body [11,12,14]. Se intake by plants is mainly from the environment [9]. For vegetables, the Se intake from the environment is minor, and Se treatment can significantly improve the Se accumulation in plants [7]. Se fortification can have a positive effect on the quality of plant food [93]. Some studies have shown that appropriate levels of Se can partially reduce chloroplast degradation and increase chlorophyll content [94,95,96]. Se treatment significantly improves the vegetative growth and photosynthetic pigment accumulation of peanut plants [94], and Se supplementation could effectively improve the antioxidant capacity of broccoli, improve growth performance and increase Se concentration [89]. In addition, eating Se-enriched foods can improve the body’s antioxidant and anti-inflammatory abilities to resist external damage [88,89,90]. Studies have shown that Se-enriched radish sprouts can inhibit inflammation and reduce cell apoptosis to alleviate liver injury induced by carbon tetrachloride [95]. Eating Se-enriched lentils can increase the excretion of heavy-metal arsenic and maintain the health level of the body [96]. The consumption of Se-enriched fruits may contribute to daily Se supplementation, thereby improving human health [13]. Se-enriched kiwifruit (Se-Kiwi) can significantly improve the activity of antioxidant enzymes in the liver of hyperlipidemia mice, reduce the content of liver fat, inhibit the accumulation of abdominal-fat cells and restore the shape of liver to a healthy level [97]. Se is usually used as a feed additive to improve feed efficiency and growth, carcass traits and the meat quality of livestock and poultry [98]. Adding a proper Se source to the feed of laying hens can increase the Se concentration in eggs and obtain Se-enriched eggs [98,99]. Dietary supplementation of Se-enriched yeast can activate glutathione and the thioredoxin system to improve meat quality and enhance the total antioxidant capacity of broilers [100,101]. Adding olive leaves supplemented with Se to a rabbit’s diet can improve the oxidation state and antioxidant content of rabbit meat [102]. It was found that eating Se-enriched pork can increase serum Se from 73.19 ± 15.68 μg.L^−1^ to 73.73 ± 15.13 μg.g L^−1^, and reduce the total cholesterol level in the human body [103]. However, it should be noted that, even though Se plays an important role in the body, an excessive Se content in the body causes Se poisoning, so the doses of Se in production and application should be strictly controlled [96,97,98,99,100,101].

### 5.2. Nano-Se Has the Potential to Be a High-Quality Se Supplement

Because traditional Se supplements usually have the disadvantages of low absorption and toxicity, it is of great significance to develop a novel Se transporter [1]. Nano-Se is zero-oxidation valence Se (Se 0). In comparison to the other oxidation states (Se + IV, Se + VI), Nano-Se has a low toxicity and excellent bioavailability [104,105]. It is also very unstable and easily converted to other forms of Se. However, its stability can be achieved by encapsulation into suitable nano-carriers, such as polysaccharides, polyphenols, proteins and microorganisms [106]. Nano-Se can be synthesized by chemical, physical and biological syntheses. The synthesis process is simple, efficient and green [104]. Nano-Se can be used as an antioxidant to resist external stimuli and promote the expression of selenoproteins [107]. Nano-Se also has antiviral and antibacterial effects and has a potential in the treatment of cancer. In addition to its direct anti-cancer effect, it can also be used as a delivery carrier for anti-cancer drugs [103], which is of great significance for the prevention of cancer. Nano-Se has a great potential as a high-quality dietary-supplement source of Se [13,108]. However, it should be noted that, although Nano-Se plays an important role in maintaining the body’s health, its possible toxicity and side effects as a nutritional supplement should be further verified.

## 6. Conclusions and Future Outlook

Se has strong biological activity and is essential to the body. Selenoproteins, as the main utilization form of Se, participate in the regulation of multiple signaling pathways to resist external stimuli. However, special attention should be paid to Se that can be used as a “double-edged sword” element; a lack or overload of Se can have adverse effects on the body. In addition, some mechanisms of Se and selenoproteins in the regulation of diseases have not been clarified. Therefore, future research should focus on the specific mechanism of Se’s participation in regulating diseases after entering the body. This paper reviewed the biological function of Se and the role of Se in the body’s health, which provides a reference for the comprehensive study of the mechanism of Se in the future.

## Abbreviations

Selenium: Se; endoplasmic reticulum: ER; selenocysteine: Sec; selenomethionine: Se-Met; GPX: glutathione peroxidase; thioredoxin reductase: TrxR; iodothyronine deiodinase: DIO; selenium phosphorylates synthetase: SPS; selenoprotein H: SELENOH; selenoprotein O: SELENOO; selenoprotein P: SELENOP; selenoprotein T: SELENOT; selenoprotein W: SELENOW; selenoprotein N: SELENON; selenoprotein M: SELENOM; selenoprotein S: SELENOS; selenoprotein I: SELENOI; Selenoprotein K: SELENOK; selenoprotein 15: 15 kDa; selenoprotein R: SELENOR; selenoprotein V: SELENOV; non-alcoholic fatty liver disease: NAFLD; hepatocellular carcinoma: HCC; Alzheimer’s disease: AD; Parkinson’s disease: PD; Huntington’s disease: HD; inflammatory bowel disease: IBD; colorectal cancer: CRC; type-3 innate lymphocytes: ILC3; helper T-cells 17: Th17; reactive oxygen species: ROS; Graves’ disease: GD; Hashimoto’s thyroiditis: HT; autoimmune thyroid diseases: AITD; lipopolysaccharide: LPS; Se-enriched kiwifruit: Se-Kiwi.
